# SARS-CoV-2/DENV co-infection: a series of cases from the Federal District, Midwestern Brazil

**DOI:** 10.1186/s12879-021-06456-2

**Published:** 2021-07-31

**Authors:** Heidi Luise Schulte, José Diego Brito-Sousa, Marcus Vinicius Guimarães Lacerda, Luciana Ansaneli Naves, Eliana Teles de Gois, Mariana Sirimarco Fernandes, Valéria Paes Lima, Carlos Henrique Reis Esselin Rassi, Clara Correia de Siracusa, Lizandra Moura Paravidine Sasaki, Selma Regina Penha Silva Cerqueira, Cleandro Pires de Albuquerque, Ana Paula Monteiro Gomides Reis, Ciro Martins Gomes, Patricia Shu Kurizky, Licia Maria Henrique da Mota, Laila Salmen Espindola

**Affiliations:** 1 Programa de Pós-Graduação em Ciências Médicas, Faculdade de Medicina, Universidade de Brasília – UnB, Brasília, Brazil; 2 Fundação de Medicina Tropical Dr Heitor Vieira Dourado, Manaus, Brazil; 3 Universidade do Estado do Amazonas, Manaus, Brazil; 4 Instituto Leônidas & Maria Deane, Fundação Oswaldo Cruz, Manaus, Brazil; 5 Hospital Universitário de Brasília, Brasília, Brazil; 6 Hospital Regional do Gama, Secretaria de Estado de Saúde do Distrito Federal, Brasília, Brazil; 7 Corpo Bombeiros Militar do Distrito Federal, Brasília, Brazil; 8 Hospital Sírio Libanês de Brasília, Brasília, Brazil; 9 Faculdade de Medicina, UNICEUB, Brasília, Brazil; 10 Programa de Pós-Graduação em Medicina Tropical, Faculdade de Medicina, Universidade de Brasília – UnB, Brasília, Brazil

**Keywords:** COVID-19, Dengue, SARS-CoV-2, DENV, Co-infection, Case series

## Abstract

**Background:**

Since the novel coronavirus disease outbreak, over 179.7 million people have been infected by SARS-CoV-2 worldwide, including the population living in dengue-endemic regions, particularly Latin America and Southeast Asia, raising concern about the impact of possible co-infections.

**Methods:**

Thirteen SARS-CoV-2/DENV co-infection cases reported in Midwestern Brazil between April and September of 2020 are described. Information was gathered from hospital medical records regarding the most relevant clinical and laboratory findings, diagnostic process, therapeutic interventions, together with clinician-assessed outcomes and follow-up.

**Results:**

Of the 13 cases, seven patients presented Acute Undifferentiated Febrile Syndrome and six had pre-existing co-morbidities, such as diabetes, hypertension and hypopituitarism. Two patients were pregnant. The most common symptoms and clinical signs reported at first evaluation were myalgia, fever and dyspnea. In six cases, the initial diagnosis was dengue fever, which delayed the diagnosis of concomitant infections. The most frequently applied therapeutic interventions were antibiotics and analgesics. In total, four patients were hospitalized. None of them were transferred to the intensive care unit or died. Clinical improvement was verified in all patients after a maximum of 21 days.

**Conclusions:**

The cases reported here highlight the challenges in differential diagnosis and the importance of considering concomitant infections, especially to improve clinical management and possible prevention measures. Failure to consider a SARS-CoV-2/DENV co-infection may impact both individual and community levels, especially in endemic areas.

## Background

Since the outbreak of the novel coronavirus disease (COVID-19), over 179.7 million people have been infected by SARS-CoV-2 in over 210 countries [1]. This includes developing regions that are endemic for dengue fever, particularly Latin America and Southeast Asia, which raised concern about the effects of co-infection with dengue viruses (DENV) and SARS-CoV-2 [2–6].

In Brazil and other tropical countries, SARS-CoV-2 was first notified during an ongoing epidemic of dengue fever, with Midwestern Brazil presenting the highest incidence nationwide [2–6]. The city of Brasilia, located in the Federal District of Brazil, alone had an incidence of 1469.8 cases per 100,000 inhabitants [7]. Dengue fever cases usually reach their peak in the first semester of the year in the Federal District, greatly influenced by precipitation during summer time [8]. Due to similarities in their epidemiological and clinical profiles, co-infections of DENV with other febrile syndromes have been reported [9, 10].

In light of the current pandemic scenario, several other infections may share an array of symptoms with COVID-19. Limited data are available in the literature regarding the SARS-CoV-2/DENV co-infection, with a single case reported in an urban area of Brazil, where the patient progressed to a favorable outcome [11]. Thus, it is necessary to understand the spectrum of this co-infection for timely diagnosis and tailored clinical management, which could prove lifesaving in severe cases. Herein, we describe a series of 13 patients with a SARS-CoV-2/DENV co-infection in Brazil aiming to disclose important details of this emerging co-infection considering the diagnosis, clinical management and possible prevention measures.

## Methods

This retrospective study evaluated 13 patients co-infected with SARS-CoV-2 and DENV diagnosed at the *Hospital Universitário de Brasília* (HUB), a university hospital located in Brasília (Federal District, Brazil), between April and September of 2020. Patients that were diagnosed with COVID-19 and dengue fever within a maximum timespan of 15 days were considered co-infected. All patients included in this study: (a) had positive RT-PCR for SARS-CoV-2, and a positive NS1 or IgM ELISA for DENV with strongly suggestive dengue symptoms in a maximum timeframe of 15 days (RT-PCR for DENV was not performed for any of the patients); (b) resided in the Federal District; (c) were over 18 years old, and (d) were able to understand the information contained in the Free and Informed Consent Form.

For all patients, SARS-CoV-2 infection was confirmed in nasopharyngeal swab samples by the reverse transcriptase-polymerase chain reaction (RT-PCR). The High Pure Viral Nucleic Acid Version 18 Kit (Roche Diagnostics®, Germany) was used for viral RNA extraction. RT-PCR was performed on a StepOnePlus™ Real-Time PCR System (Applied Biosystems®, USA) using the Molecular SARS-CoV-2 (E/RP) - Bio-Manguinhos kit (Rio de Janeiro, Brazil), according to the manufacturer’s instructions. DENV infection was confirmed by either NS1 or IgM, as described in Table 1. Detection of DENV NS1 antigen was performed using a rapid immunochromatographic test (ABBOTT-Alere® S.A., Brazil), following the manufacturer’s instructions. Antibodies for DENV were detected by a commercially available indirect IgM enzyme-linked immunosorbent assay (ELISA) (Euroimmun®, Germany).

**Table 1:** Clinical and laboratory characteristics of COVID-19/dengue cases

Case	Sex	Clinical presentation	COVID-19 diagnosis/ date	Dengue diagnosis/date	Concomitant condition	Hospitalized	Platelet count (/μL)	Lymphocyte count (/μL)	Main signs and symptoms at first evaluation
1	M	AUFS	RT-PCR+ 15/04/2020	NS1+ 05/04/2020	Diabetes Hypertension	No	84,000	2982	fever, myalgia, ecchymosis dyspnea (SpO_2_ = 95%)
2	F	ARFS	RT-PCR + 22/04/2020	NS1 + 20/04/2020	No	Yes	93,000	730	fever, dry cough, dyspnea, myalgia
3	F	ARFS	RT-PCR + 15/04/2020	IgM + 15/04/2020	Diabetes	Yes	169,000	2627	dyspnea (SpO_2_ = 91%)
4	M	ARFS	RT-PCR+ 05/05/2020	NS1+ 30/04/2020	Hypopituitarism Adrenal insufficiency	No	110,000	3254	myalgia, ecchymosis dyspnea
5	F	AUFS	RT-PCR + 08/05/2020	IgM + 05/05/2020	Pregnancy	Yes	94,000	1500	retro-orbital pain, arthralgia, myalgia
6	M	AUFS	RT-PCR + 26/06/2020	NS1 + 26/06/2020	No	No	–	–	fever
7	M	ARFS	RT-PCR + 18/07/2020	IgM + 18/07/2020	-*	No	191,000	2200	dry cough, sore throat
8	F	ARFS	RT-PCR + 17/07/2020	IgM + 23/07/2020	No	No	238,000	1490	myalgia, nasal congestion, dyspnea, fatigue, diarrhea
9	M	AUFS	RT-PCR + 12/07/2020	IgM + 12/07/2020	No	No	180,000	1561	retro-orbital pain, myalgia, fever, anosmia, diarrhea
10	F	AUFS	RT-PCR + 22/07/2020	IgM + 22/07/2020	Pregnancy Gestational diabetes Chronic gastritis Depression	No	196,000	1500	fever, dry cough, myalgia, sore throat, nasal congestion, diarrhea, anosmia, ageusia, pruritus
11	F	AUFS	RT-PCR + 06/08/2020	NS1 + 30/07/2021	Pituitary tumor Hypopituitarism	No	50,000	3100	fever, myalgia and fatigue dyspnea (SpO_2_ = 93%)
12	M	AUFS	RT-PCR + 22/08/2020	IgM + 22/08/2020	No	No	169,000	2327	myalgia
13	F	ARFS	RT-PCR+ 24/09/2020	NS1+ 12/09/2020	No	Yes	87,000	1450	fever, myalgia dyspnea (SpO_2_ = 92%) urethral bleeding

*ARFS* Acute Respiratory Febrile Syndrome; *AUFS* Acute Undifferentiated Febrile Syndrome; *RT-PCR* reverse transcriptase polymerase chain reaction; *IgM* immunoglobulin M; *NS1* Non-structural protein 1. *- = information not available

The cases were reported by a team of healthcare professionals in the COVID-19 and endemic diseases co-infections study approved by Brazil’s National Committee of Ethics under CAAE 34164820.6.0000.0030. All patients received clarification about the general proposal of the study both orally and in writing, based on the printed text of the Free and Informed Consent Form (FICF), which was signed by all patients included in this study. The FICF also assured patients of confidentiality regarding their names and personal data, as well as the possibility of waiving their participation at any time.

Information collected from hospital medical records included: 1) most relevant clinical and laboratory findings, such as clinical presentation (Acute Respiratory Febrile Syndrome - ARFS, or Acute Undifferentiated Febrile Syndrome - AUFS); 2) concomitant conditions; 3) need for hospitalization; 4) platelet count; 5) lymphocyte count, and finally 6) main signs and symptoms at first evaluation.

In addition, information was gathered about the diagnostic process, from the initial to the final diagnosis of a concomitant infection with SARS-CoV-2 and DENV, including: an eventual delay between first and final diagnosis; description of the therapeutic interventions, together with pharmacological, preventative and self-care measures; clinician-assessed outcomes, and follow-up. This descriptive study numbered the reported cases from 1 to 13 to ensure de-identification of patient-specific information.

## Results

**Fig. 1:**
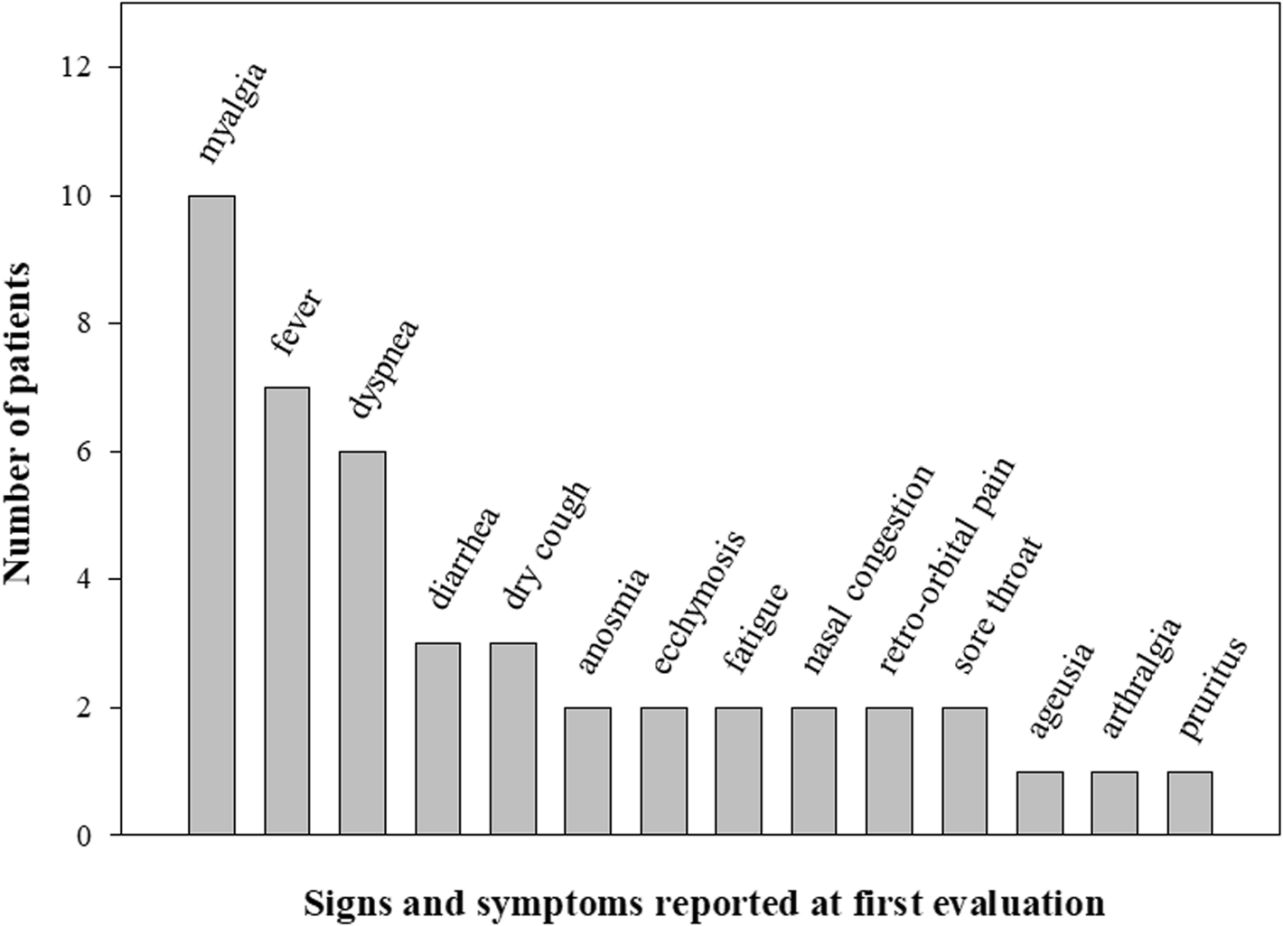
Signs and symptoms described in hospital medical records at the first evaluation of patients with SARS-CoV-2/DENV co-infection

Of the 13 SARS-CoV-2/DENV co-infection cases reported in this study, with ages ranging between 27 and 79, seven patients were female, and seven patients presented AUFS. Pre-existing morbidities were present in four patients, including diabetes in one patient, diabetes and hypertension in one patient, hypopituitarism and pituitary tumor in one patient, and hypopituitarism and adrenal insufficiency in another patient. Moreover, two patients were pregnant. One of the pregnant patients (Case 5) gave birth without any complications during the course of the SARS-CoV-2/DENV co-infection. Patient case number 10, also pregnant, presented gestational diabetes, chronic gastritis and depression, but experienced no pregnancy-related complications until her 10th week of pregnancy. A total of six patients presented thrombocytopenia, and only one patient had lymphopenia. The most frequently reported symptoms at first evaluation were myalgia, fever and dyspnea, followed by dry cough and diarrhea, as presented in Fig. 1. The clinical and laboratory aspects of all 13 patients are summarized in Table 1.

**Fig. 2:**
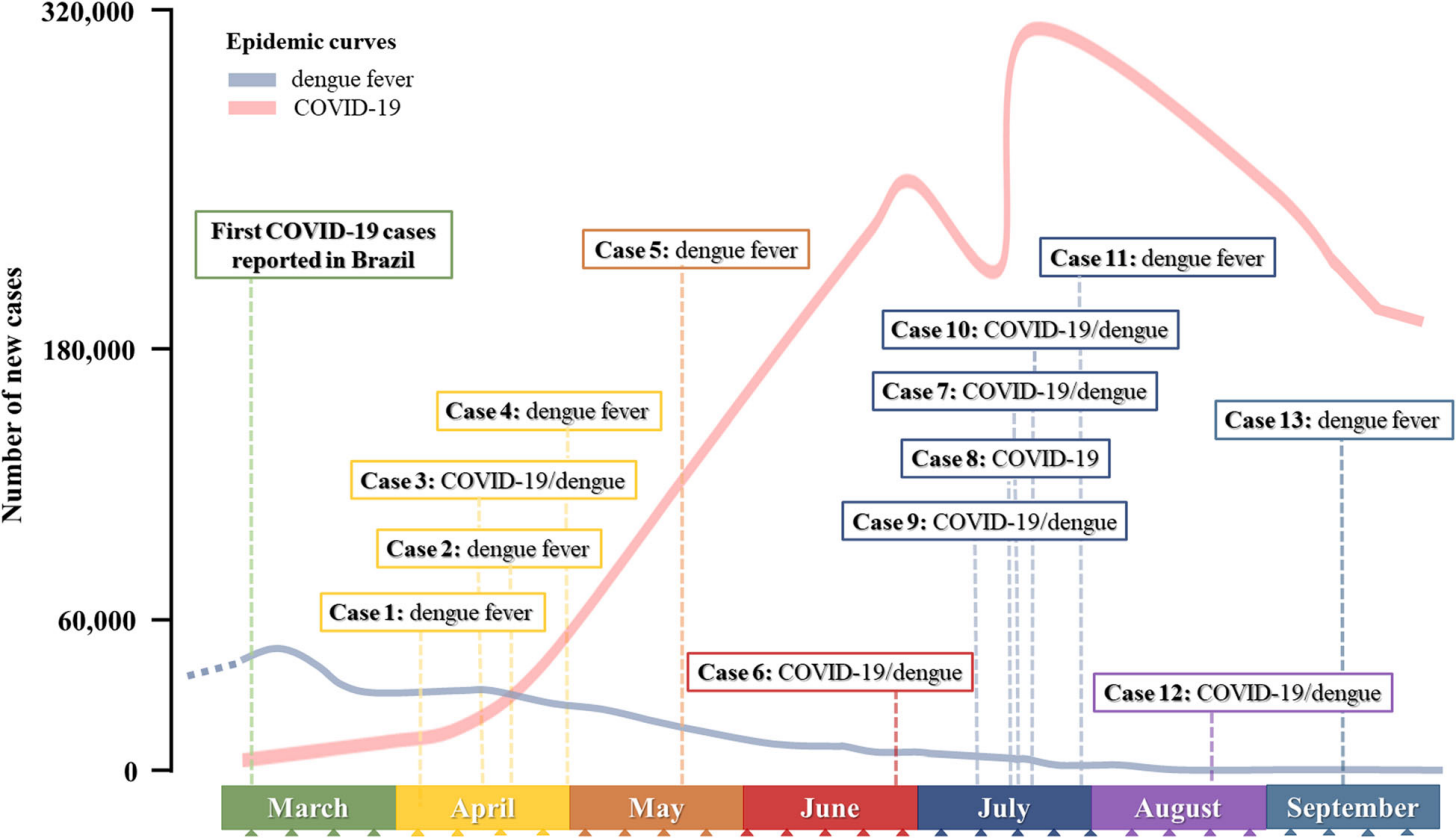
Timeline presenting the initial diagnosis established in the 13 SARS-CoV-2/DENV co-infection cases reported by the *Hospital Universitário de Brasília* since the beginning of the COVID-19 pandemic in Brazil. The x-axis divisions represent weeks. Background lines represent the epidemic curves for dengue fever (blue line) and COVID-19 (red line), based on official reports from the Brazilian Ministry of Health [7, 12]

As displayed in Table 2, the initial diagnosis for six of the 13 patients was dengue fever, and only after a delayed period, ranging from 2 to 12 days, were these patients also diagnosed with COVID-19. Suspicion of co-infection with SARS-CoV-2 in these patients was raised mainly due to the persistence or emergence of fever or respiratory symptoms. Conversely, in one case, the patient was first diagnosed with COVID-19, and 6 days later also with dengue fever. In six cases, the initial diagnosis was SARS-CoV-2/DENV co-infection. These patients were initially diagnosed with both infections due to previous observations of co-infection cases in the HUB hospital. For the Case1 patient, the initial hypothesis was actually a reaction to the flu vaccine, however, the presence of ecchymosis justified further investigation which led to dengue fever diagnosis. The timeline for variations in initial diagnosis from April to September 2020 are shown in Fig. 2. Four of the six cases initially diagnosed as dengue occurred in the first 2 months of the pandemic (Fig. 2).

**Table 2:** Initial diagnosis; delay between first diagnosis and final diagnosis of co-infection; therapeutic intervention; outcomes and follow-up of COVID-19/dengue cases

Case	Sex	Initial diagnosis	Delay	Therapeutic intervention	Outcome and follow-up
1	M	Dengue fever	10 days	Analgesics	Clinical improvement in 10 days
2	F	Dengue fever	2 days	Analgesics Hydration with 0.9% saline Amoxicillin Clavulanate Prophylaxis of thrombosis with compression stockings	7 days in hospital; no follow-up
3	F	COVID-19/dengue	None	Analgesics Enoxaparin 40 mg/day	4 days in hospital; no follow-up
4	M	Dengue fever	5 days	Analgesics Prednisone dose was increased from 5 to 15 mg for 5 days, to avoid adrenal insufficiency	Clinical improvement in 15 days
5	F	Dengue fever	3 days	Hydroxychloroquine 400 mg 2x/day for 1 day Chloroquine 450 mg for 1 day Enoxaparin 40 mg/day for 2 weeks Azithromycin 500 mg/day for 5 days Ceftriaxone 2 g/day for 5 days	2 hospitalizations (due to dengue symptoms, and later, due to childbirth) Clinical improvement in 13 days
6	M	COVID-19/dengue	None	Self-medication with ivermectin (6 mg/kg)	Clinical improvement in 4 days
7	M	COVID-19/dengue	None	Analgesics Hydration with 0.9% saline	Clinical improvement in 14 days
8	F	COVID-19	6 days	Azithromycin (500 mg) for 2 days Self-medication with ivermectin (6 mg/kg)	–
9	M	COVID-19/dengue	None	Analgesics	Clinical improvement in 4 days
10	F	COVID-19/dengue	None	Analgesics Prednisone 20 mg for 5 days Hydration with 0.9% saline	Clinical improvement in 21 days
11	F	Dengue fever	7 days	Azithromycin (500 mg) for 5 days Prednisone dosage increased from 5 to 20 mg for 7 days	Pulmonary resolution in 15 days (patient had 30% of lung commitment)
12	M	COVID-19/dengue	None	None	Clinical improvement in 7 days
13	F	Dengue fever	12 days	Corticoids, azithromycin (500 mg) Oxygen in the first 2 days	5 days in hospital; clinical improvement in 20 days

*- = information not available

Antibiotics were used as part of the therapeutic intervention in five cases; anticoagulants were used in two cases; analgesics were used in eight cases; prednisone in one case, and hydroxychloroquine/chloroquine in one case. The prednisone dosage was increased for the two patients with pre-existing hypopituitarism. Additionally, two patients self-medicated with ivermectin. Non-pharmacological measures were also applied, such as hydration with 0.9% sodium chloride infusion in three cases and oxygen in one patient. In total, four patients were hospitalized. None of the patients presented dengue fever with warning signs or severe dengue, and none of them were transferred to an intensive care unit or died. Clinical improvement was verified in all patients after a maximum of 21 days. Table 2 summarizes diagnosis, therapeutic interventions, outcomes and follow-up information for each of the 13 cases.

## Discussion

The COVID-19 pandemic has had a severe impact with millions of deaths worldwide [1]. In dengue-endemic regions, the situation is aggravated by socio-economic aspects and the occurrence of epidemic arboviruses [3]. In addition to the first case reported in Brazil, also in the Federal District [11], SARS-CoV-2/DENV co-infection cases were reported in other tropical and developing countries, where these infections are also considered a defying public health problem [13–15]. The co-infection cases described to date, together with the 13 cases reported herein, highlight the difficulty in reaching the final diagnosis, since both diseases share similar signs, symptoms and laboratory features. These obstacles in the diagnostic process can be detrimental to the patient’s condition and increase the burden on the healthcare system, especially in the context of the COVID-19 pandemic, as these are diseases with substantial morbidity and mortality [3].

The precise diagnosis of COVID-19 alone is already a significant challenge in clinical practice as the differential diagnosis with influenza and other respiratory infections must be considered. This problem is reflected in the number of deaths by respiratory diseases reported between March and November of 2020 in Brazil, where a 1225% increase in acute respiratory distress syndrome (ARDS) and a 40% increase in undetermined deaths (deaths linked to respiratory diseases, but not conclusive) can be seen when compared to the same period in 2019 [16]. In this sense, other endemic febrile diseases complicate the clinical picture even further.

The differential diagnosis problem with febrile diseases becomes evident after analyzing the cases reported here, where the similarities between symptoms in the initial stages of COVID-19 and dengue fever, or asymptomatic presentation of one of them, delayed the diagnosis of concomitant infections (Table 2). Even retro-orbital pain, a symptom usually associated with dengue, is being commonly reported in COVID-19 cases [17]. In the context of this case series, at the start of the pandemic dengue fever was usually the first diagnosis contemplated by physicians (Fig. 2), and co-infection with SARS-CoV-2 was, therefore, determined with delay. Fortunately, during the course of the pandemic, healthcare professionals started to consider the possibility of concomitant infections in the initial diagnosis, as highlighted in Fig. 2.

As dengue fever and COVID-19 require different clinical management, incorrect or delayed diagnosis can have serious consequences [11]. The use of anticoagulants is especially concerning since they are frequently used in COVID-19 patients to protect against thrombotic events, but should be avoided in all patients with dengue fever, as they can increase the risk of thrombocytopenia and even trigger Reyes syndrome, a rare condition characterized by hepatitis and encephalopathy [17]. Furthermore, several factors, such as hypertension, diabetes, obesity and old age, that are associated with poor prognosis in COVID-19 [18], may also complicate dengue fever. Some of these factors are present in the co-infected patients reported here, and in previously reported cases [15].

Some clinical and epidemiological clues can aid differential diagnosis. Generally, the seasonality is opposite: respiratory infections are usually reported in winter whereas dengue is usually in summer. However, COVID-19 cases in Brazil do not follow a clear seasonal pattern, as commonly observed for influenza and other respiratory viruses. It is not yet clear whether COVID-19 will become seasonal or will continue to spread throughout the year, especially because of its stability in comparison to other respiratory viruses (such as influenza) and the presence of an immunologically-susceptible population [19]. Meanwhile, public health policies and individual countermeasures are in place to mitigate the spread.

The usual findings of thrombocytopenia and lymphopenia are common in both diseases [20, 21]. However, cavitary effusions and bleeding are not commonly observed in COVID-19, which should raise suspicion, even with pulmonary clear ground-glass opacities typical of such infection. The diagnosis of COVID-19 during a pandemic might impact and lead to a reduction in the number of dengue cases diagnosed, contributing to underdiagnosis and delayed fluid interventions, which are lifesaving in severe dengue cases [22]. Official reports issued by the Brazilian Ministry of Health showed a decrease in the number of dengue cases and deaths compared to 2019, possibly fueled by resource and personnel allocation to SARS-CoV-2 diagnosis in Brazil [7], which could have led not only to under-reporting of cases and deterioration in surveillance, but also a worsening in control interventions [2].

An additional limitation is mainly related to the serological response of these infections. The low specificity of some rapid tests and commercial kits designed to detect IgM antibody for DENV cross-reactivity must be considered [23]. In addition, the possibility of a hospital-acquired SARS-CoV-2 infection is also a factor to be taken into consideration in patients hospitalized due to severe dengue.

Regarding cross-reactivity, confirmatory diagnosis via RT-PCR for DENV would be ideal. However, in the context of clinical practice, confirmation by RT-PCR is frequently not the most common approach as clinical management of dengue fever is decided primarily on signs and symptoms [24]. IgM ELISA or NS1 tests are often preferred as they are more available, and more affordable, in dengue-endemic regions [25–27]. In addition, the serological sample collection time offers more flexibility, while the higher stability of immunoglobulins facilitates transportation [26].

In addition, although it is known that clinical manifestations can vary according to serotype [28], in the Federal District, RT-PCR results are only released in up to 12 working days [29], by which time the symptoms have already subsided in the majority of cases [24]. Therefore, health professionals often use serological tests to confirm dengue diagnosis. That said, information about the circulating serotypes can be obtained from the weekly regional epidemiological bulletins that register the infrequently reported serotype identification. Between January and September of 2020, these bulletins reported 312 DENV-1 and 16 DENV-2 serotype identifications in the residencial areas of the patients included in the present study [7]. Nonetheless, lack of identification of the dengue serotype is a limitation of this study as it has clinical and epidemiological significance [28]. An experimental design incorporating the RT-PCR test for DENV should be considered for future studies.

## Conclusions

This study presents a detailed case series of SARS-CoV-2/DENV co-infection in the Federal District, Midwestern Brazil. Despite being limited by a retrospective study design, a reduced number of cases, restricted testing capacity of the Brazilian healthcare system and some unavailable data, this case series is a source of valuable information that is currently missing in the literature. Our study demonstrates that failure to consider a SARS-CoV-2/DENV co-infection may impact both individual and community levels, especially in endemic areas. Other vector-borne infections such as chikungunya, Zika and malaria are highly prevalent in many tropical areas, and how respective co-infections with COVID-19 impact lethality requires further observational studies. Both diseases could be more lethal among more vulnerable populations who have less access to a high-quality health system, despite the universal incidence in all social levels. Therefore, the constant gathering of information and discussion about co-infections is crucial to improve diagnosis, clinical management and prevention measures.

## Data Availability

The datasets generated and/or analyzed during the current study are not publicly available so as to not compromise patient anonymity. However, this data is available from the corresponding author upon reasonable request.

## Abbreviations

COVID-19: Coronavirus disease-2019
SARS-CoV-2: Severe Acute Respiratory Syndrome Coronavirus 2
DENV: Dengue virus
RT-PCR: Reverse transcriptase polymerase chain reaction
NS1: Non-structural protein 1
IgM: Immunoglobulin M
ARFS: Acute Respiratory Febrile Syndrome
AUFS: Acute Undifferentiated Febrile Syndrome
